# Potential Pharmaceutical Applications of Quercetin in Cardiovascular Diseases

**DOI:** 10.3390/ph15081019

**Published:** 2022-08-18

**Authors:** Paraskevi Papakyriakopoulou, Nikolaos Velidakis, Elina Khattab, Georgia Valsami, Ioannis Korakianitis, Nikolaos PE Kadoglou

**Affiliations:** 1 Laboratory of Biopharmaceutics-Pharmacokinetics, Department of Pharmacy, School of Health Sciences, National & Kapodistrian University of Athens, 15784 Athens, Greece; 2 Medical School, University of Cyprus, Nicosia 2029, Cyprus

**Keywords:** quercetin, cardiovascular diseases, diabetes mellitus, hypertension, dyslipidemia

## Abstract

Quercetin, as a member of flavonoids, has emerged as a potential therapeutic agent in cardiovascular diseases (CVDs) in recent decades. In this comprehensive literature review, our goal was a critical appraisal of the pathophysiological mechanisms of quercetin in relation to the classical cardiovascular risk factors (e.g., hyperlipidemia), atherosclerosis, etc. We also assessed experimental and clinical data about its potential application in CVDs. Experimental studies including both in vitro methods and in vivo animal models mainly outline the following effects of quercetin: (1) antihypertensive, (2) hypolipidemic, (3) hypoglycemic, (4) anti-atherosclerotic, and (5) cardioprotective (suppressed cardiotoxicity). From the clinical point of view, there are human studies and meta-analyses implicating its beneficial effects on glycemic and lipid parameters. In contrast, other human studies failed to demonstrate consistent favorable effects of quercetin on other cardiometabolic risk factors such as MS, obesity, and hypertension, underlying the need for further investigation. Analyzing the reason of this inconsistency, we identified significant drawbacks in the clinical trials’ design, while the absence of pharmacokinetic/pharmacodynamic tests prior to the studies attenuated the power of clinical results. Therefore, additional well-designed preclinical and clinical studies are required to examine the therapeutic mechanisms and clinical efficacy of quercetin in CVDs.

## 1. Introduction

**Figure 1:**
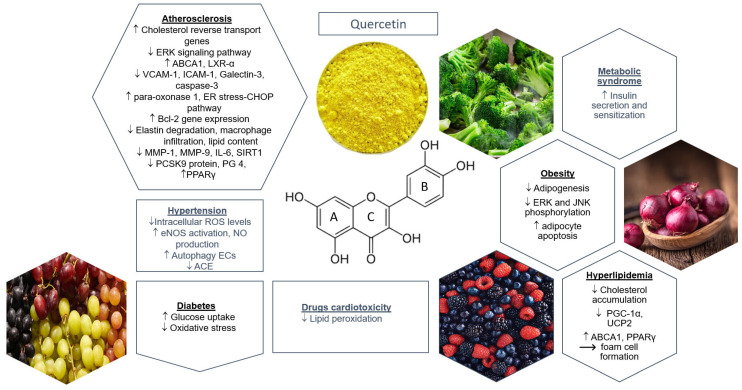
Schematic representation of the most important cardiovascular protective properties of quercetin and the related possible mechanism of action. Arrows indicate increase (↑) or decrease (↓) of the respective biomarker expression and the resulting formation (→). Key: ABCA1: ATP-binding cassette transporter A1; ACE: angiotensin-converting enzyme; Bcl-2: B cell lymphoma-2; CHOP: C/EBP homologous protein; ECs: endothelial cells; eNOS: endothelial NO synthase; ER: endoplasmic reticulum; ERK: extracellular signal-regulated kinase; ICAM-1: intercellular adhesion molecule 1; IL-6: interleukin 6; JNK: Jun N-terminal kinase; LXR-α: liver X receptor; MMP: matrix metalloproteinase; PCSK9: proprotein convertase subtilisin/kexin type 9; PG: prostaglandin; PGC-1α: peroxisome proliferator-activated receptor γ coactivator-1α; PPARγ: peroxisome proliferator-activated receptor γ; ROS: reactive oxygen species; SIRT1: sirtuin 1; UCP2: uncoupling protein 2.

Flavonol quercetin has recently received extensive attention from the medical and research communities because of its significant potential therapeutical applications, including antioxidant, antitumor, antiatherogenic, antithrombotic, and cardioprotective properties [1,2,3]. In 1930, rutin (quercetin 3-O-rutinoside), one of quercetin’s most common forms, was isolated from oranges, and it was initially believed to be a vitamin; for a while, rutin was called vitamin P [4]. Quercetin, from Latin *quercetum* (oak forest), was first described in 1936 [5,6]. It is a low-cost substance found widely in many plant species [1]. Fruits, vegetables, tea, and red wine are the major sources of flavonols. However, some characteristics of the molecule (Figure 1), including low water solubility, poor bioavailability, instability in the biological environment, and enzymatic degradation with very small half-life, have largely prevented its clinical usage [2,3]. Trying to overcome those drawbacks, other studies examined its binding ability with iron [7] and copper [8] to improve its bioavailability.

Recently, preventive and therapeutic effects of flavonoids on the cardiovascular system, alone or in combination with other agents, have been outlined in a plethora of studies [3]. The first randomized controlled trials (RCTs) examining the cardioprotective effects of quercetin on healthy subjects were conducted in 1998 [9,10]. Subsequently, a growing body of evidence has supported the clinical importance of modified dosage forms of quercetin, using other carriers such as nanoparticles and microemulsions [2,11].

The aim of this comprehensive review was to summarize and critically discuss the main pharmacological mechanisms of quercetin in relation to atherosclerosis and the classical cardiovascular risk factors such as diabetes mellitus (DM), hyperlipidemia, etc. We also tried to assess the potential application (protective and/or therapeutic) in cardiovascular diseases (CVDs) based on experimental and clinical studies. The main cardiovascular protective properties of quercetin are depicted with the possible mechanisms of action in Figure 1.

## 2. Literature Search Strategy

For the needs of this review paper, we searched MEDLINE and EMBASE, Web of Science, Cochrane, and Google Scholar databases for English-language publications from 1990 to May 2022. We also checked the reference lists of the identified articles to find any additional relevant articles. Our search included the titles, abstracts, and medical subject headings (MeSH), and we used the following search terms: quercetin, atherosclerosis, diabetes mellitus, hyperglycemia, hypertension, hyperlipidemia, metabolic syndrome, cardiovascular diseases, coronary artery disease. Four investigators (N.B., C.P., M.V., and P.P.) performed the literature search independently. In our search, we included both in vitro and in vivo preclinical experimental studies, as well as clinical studies (both clinical investigations and clinical meta-analyses). To draw firm conclusions, we excluded the following studies: full text unavailable, publication language other than English, conference abstracts, and mixing of quercetin and other substances in interventional arms.

Using the abovementioned terms, we initially found 2120 hits. After the abstracts’ screening, we removed 192 duplicated studies, and also 1865 irrelevant studies; 110 full-text studies were screened for eligibility. After removing the studies with wrong design, irrelevant outcomes, unavailable full text, we ended up with a total of 30 experimental studies, 10 systematic reviews and meta-analyses of clinical data, and 20 additional clinical studies which were not included in those meta-analyses.

## 3. Results and Discussion

### 3.1. Chemical Structure of Quercetin and Its Main Derivatives

**Figure 2:**
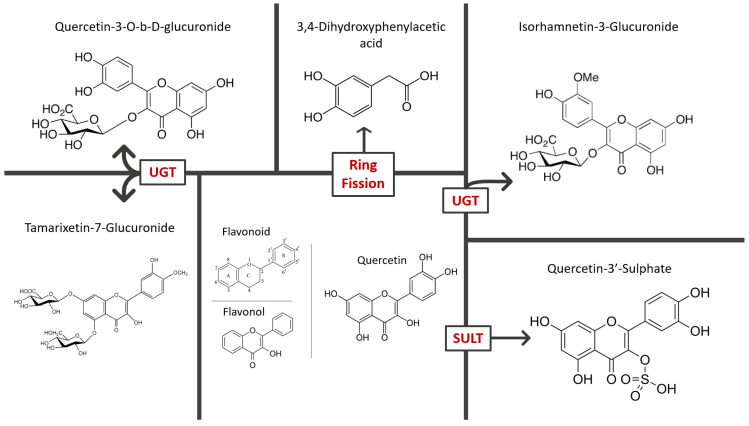
Chemical structure of quercetin and its main derivatives (metabolites).

Flavonoids are a group of different phytonutrients with variable phenolic structures found in almost all plants [12]. These natural low-molecular-weight substances are secondary metabolites characterized by a polyphenolic chemical structure [4,12]. The main feature of all flavonoids is the basic flavan skeleton: a 15-carbon phenylpropanoid chain, C6–C3–C6, which consists of two aromatic rings (A and B) and a heterocyclic pyrane ring (C) [13] (Figure 1). The different subgroups of flavonoids result from the carbon attached between the two rings (B and C) of the molecule and the degree of unsaturation and oxidation of the C ring [3,12] (Figure 2). Flavonoids can be subdivided into anthocyanins, chalcones, flavanones, flavones, flavonols, and isoflavonoids [3,13]. Quercetin (3,3′,4′,5,7-pentahydroxyflavone) belongs to flavonols with a ketone group [12] (Figure 1). Flavonols are characterized by an unsaturated C ring at the C2–C3 position, which can be hydroxylated at C3 and oxidized at C4 [13]. There are variable methylation, hydroxylation, and glycosylation patterns leading to the formation of quercetin derivatives (Figure 2). Glycosylation may occur at any of the five hydroxyl groups of quercetin, but most commonly occurs at the 3-OH group [1,12,14,15]. Quercetin has strong antioxidant properties because of its chemical structure, including its highly reactive hydroxyl groups, catechol functionality (ortho-dihydroxyl) on the B ring, and a Δ^2^ double bond adjacent to the 4-oxo group in the C ring [16]. Studies have shown that quercetin can exist conjugated with carbohydrates, lipids, alcohol, and sulfate group or unconjugated as an aglycone [16]. Although quercetin metabolites have fewer antioxidant capabilities, there are some metabolic derivatives of quercetin which effectively remove reactive species of the body [16].

### 3.2. Quercetin’s Metabolic Pathways

*Per os* administration of quercetin is slightly absorbed in the stomach, followed by extensive intestinal absorption via the Na^+^-dependent cotransporter (SGLT1) and the first pass metabolism [17,18]. During metabolic phase II, quercetin is conjugated in the small intestine via glucuronidation, sulphuration, and methylation as it is confirmed by the presence of the corresponding metabolites in human small intestine microsomes [19]. Several enzymes are responsible for the catalysis of conjugation reactions such as sulfotransferases (SULTs), uridine-5′-diphosphate glucuronosyl transferases (UGTs), and catechol-*O*-methyl- transferases (COMTs). Namely, UGT-mediated glucuronidation is required for the production of the active metabolite quercetin-3-O-b-D-glucuronide, as well as of tamarixetin-7-glucuronide and isorhamnetin-3-glucuronide. Moreover, the sulphuration regulated by SULTs lead to the metabolite quercetin-3′-sulphate, while ring fission—to 3,4-dihydroxyphenylacetic acid [20]. Most of the metabolites in the bloodstream are glucurono–sulfo-conjugates of isorhamnetin and quercetin, while a minor part are quercetin glucuronides and methoxylated forms [21]. However, oral administration of quercetin in rats has shown that quercetin-3-O-b-D-glucuronide is the major bioactive metabolite in plasma [22]. Moreover, tamarixetin-7-glucuronide and isorhamnetin-3-glucuronide are accumulated in tissues, and they have been associated with anti-inflammatory and anti-atherosclerotic effects, respectively [23,24]. The half-life of quercetin metabolites shows a range of 11 to 28 h, which is considered high compared to other phytochemicals [18]. These metabolites are released into the blood and lymphatic circulation, as evidenced by the presence of quercetin glycosides in both the portal vein and lymph, while quercetin levels have been found below the detection limits or equal to 6.7% of the administered dose [17,25]. Subsequently, the metabolites reach the liver and enter hepatocytes via either passive diffusion or through organic anion transporters (OAT) and the organic anion transport protein (OATP) [26]. After hepatic uptake, they are further metabolized by phase II conjugating enzymes and pass into the circulation or are localized in biliary excretion [25]. Quercetin is excreted with feces and urine, while there are reports of lung elimination when the flavonoid is administered in high doses [18].

The limited absorption due to the low solubility of the compound in gastrointestinal fluids and the extensive metabolism into inactive metabolites that are excreted rapidly from the body contribute to quercetin’s low oral bioavailability. It is worth mentioning that the bioavailability has not yet been accurately determined due to the high variability of the reported pharmacokinetic parameters even after the consumption of the same dose and form of quercetin. This fact is likely attributed to the interindividual variability of β-glucosidase activity, as well as variations in the performance of metabolic enzymes [20].

### 3.3. Quercetin and Cardiovascular Prevention Based on Preclinical Studies

#### 3.3.1. Hypertension

The mechanism of hypertension development has not been clearly understood. However, it is strongly associated with the vascular endothelial dysfunction [27]. Moreover, oxidative stress accompanied by reactive oxygen species (ROS) contribute to this dysfunction uncoupling endothelial nitric oxide synthase (eNOS) and reducing the nitric oxide (NO) bioavailability [28]. NO acts as an endogenous relaxation factor that regulates the vascular tone, as well as cardiac and vascular remodeling. The reduction of NO levels is related to hypoxia and cardiovascular diseases progression in patients with already existing vascular dysfunction, while its contribution to vasodilation of healthy subjects is ambiguous [29].

The protective effect of quercetin on endothelial dysfunction has been proposed to be related to its antihypertensive effect. This effect is assisted by its scavenging activity against ROS, leading to the reduction of endoplasmic stress [7]. An in vitro study has proven that 20 μM of quercetin can decrease intracellular ROS levels in endothelial cells of mesenteric arteries isolated from both hypertensive and normotensive animals [30]. Furthermore, *ex vivo* endothelial functional studies showed that quercetin can improve the vascular function via the activated protein kinase (AMPK) pathway inducing eNOS activation and, consequently, NO production [31] (Shen et al., 2012). *In vitro* studies by both Pereira et al. (2018) [32] and Lin et al. (2020) [30] support the involvement of quercetin-induced autophagy, which improves the quality of endothelial cells and NO production. Tumor studies also confirm the ability of quercetin to act as an inducer of autophagy [33,34,35]. Quercetin as a flavonoid compound of *Ugni molinae* Turcz. (murtilla) fruits has shown a vasodilatory and potentially hypotensive effect via calcium-dependent potassium channels [36]. Notably, quercetin may selectively stimulate vasorelaxant K_Ca_1.1 channels and concomitantly inhibit Ca channels’ activity with potential blood pressure-lowering impact [37].

Furthermore, quercetin is correlated with the reduction of oxidative stress in aortas of 2K1C rats. However, its effect on systolic blood pressure (SBP) values is controversial in the case of 2K1C rats [32], while in SHRs, a dose-dependent decrease is observed. In particular, a high dose of quercetin (>7 mg/kg) is required to obtain a significant decrease (*p* < 0.05) in both systolic and diastolic blood pressure (DBP) [38,39] (Elbarbry et al., 2020; Duarte et al., 2001). Notably, SBP decrease was observed either in adult (12 weeks) or young hypertensive rats (5 weeks) [30,38]. In contrast, Carlstrom et al. (2007) [40] failed to show the suppressive effects of a quercetin-supplemented diet on arterial hypertension and its complications, such as cardiac hypertrophy, vascular dysfunction, vascular remodeling, in SHRs, but they attributed this discrepancy to the mode of administration.

In several in vitro and in vivo studies, quercetin is proposed to inhibit the angiotensin-converting enzyme (ACE) by binding a zinc molecule at the active site of the enzyme and via this mechanism slows down the conversion of angiotensin I to angiotensin II [41,42]. This effect is attributed to the chemical structure of flavonoids and mainly in the presence of the double bond and the ketone group at the C ring, as well as of the 3′,4′-catechol group in the B ring (Figure 1) [43].

#### 3.3.2. Diabetes

Diabetes is a heterogeneous endocrine disorder characterized by persistent hyperglycemia due to inadequate insulin secretion or insulin resistance. Several in vitro and in vivo studies have attempted to prove the beneficial effect of flavonoids and quercetin specifically on the prevention and nutritional management of diabetes [44,45,46,47,48,49] (Table 1). Namely, studies on C2C12 and L6 skeletal muscle cell lines showed that quercetin and its most active glycosides (quercetin-3-O-glycosides) can increase the glucose uptake via a mechanism involving AMPK, stimulating the glucose transporter type 4 (GLUT4) translocation and its expression in skeletal muscles [47,50] (*p* < 0.05). Those findings are in line with those of Jiang et al. (2019) [51] where treatment of L6 myoblasts derived from rat skeletal muscles with quercetin and its glycoside isorhamnetin promoted glucose uptake. Furthermore, in vitro and in vivo experiments in the same cell line and in alloxan-induced type 2 diabetic mice, respectively, demonstrated that quercetin can reduce oxidative stress induced by ROS in various concentrations (1 μM, 10 μM, 100 μM). Moreover, quercetin may reduce malonaldehyde (MDA) levels, an index of lipid peroxidation (*p* ≤ 0.05) [45,49]. Notably, in the same study [33], 3-week-long treatment of mice with 20 mg/kg/day significantly lowered the fasting blood glucose levels (*p* ≤ 0.05), which was accompanied by the recovery of the activity of antioxidant enzymes (*p* ≤ 0.05).

In their systematic review, Shi et al. (2019) [52] summarized the antidiabetic effects of quercetin in diabetic animal models employed in several studies. Based on their analysis, quercetin significantly decreased blood glucose levels when administered intraperitoneally or orally at various doses ranging from 2.5 mg/kg/day to 200 mg/kg/day for a time period varying from 2 to 10 weeks.

#### 3.3.3. Hyperlipidemia

Hyperlipidemia constitutes one of the major risk factors for the occurrence of CVDs. Quercetin is a fundamental flavonoid of the human diet capable of reversing the effects of hyperlipidemia. In rabbits with diet-induced hyperlipidemia, quercetin reduced the serum triglycerides and cholesterol levels in those receiving a high-fat diet for 12 weeks and, to a lesser extent, in those receiving a high-fat diet for 4 weeks with a carotid artery injury. Both animal models presented a reduction in atherosclerotic lesions (aortic atherosclerosis and injured carotid artery, respectively) due to the antioxidant properties and the anti-inflammatory activity of the flavonoid [53]. Furthermore, quercetin exerted a protective effect against cardiac diastolic dysfunction induced by a high-cholesterol diet in hyperglycemic rats. This effect is expressed via the impediment of cholesterol accumulation and ATP reduction preventing the alteration of PGC-1*α*, UCP2, and PPAR*γ* receptors’ expression [54]. An in vitro study on human THP-1 derived macrophage cells showed that quercetin causes an outflow of cholesterol and decreases foam cells formation, which potentially halt the progression of atherosclerosis. This effect of quercetin is mediated via the upregulation of the ABCA1 cholesterol transporter and the PPARγ transcription factor [55]. The same findings were also reported by Cui et al. (2017) [56] after an 8-week-long treatment of apolipoprotein E-deficient atherosclerotic mice with quercetin. Recently, in vitro and in silico studies have proven that quercetin could prevent overexpression of the ICAM-1 and MCP-1 genes and impede cell migration in atherosclerotic plaques, reducing the risk of stroke [57]. In their studies, Liang et al. (2021) [58] underlined the importance of the development of novel delivery systems for quercetin solubility enhancement in both in vitro cell lines and an in vivo animal model.

#### 3.3.4. Quercetin and Cardiac Protection: Cardiomyopathies, Cardiotoxicity, Myocardial Perfusion Injury, Myocardial Infarction

##### Cardiac Injury

The vasculoprotective effects of quercetin have involved several mechanisms and different signaling pathways, such as intracellular protein kinase cascades, the reduction of oxidative stress, the inhibition of cell migration towards atherosclerotic plaques, etc. Furthermore, quercetin has been characterized as a cardioprotective agent in different kinds of cardiac injury or drug-induced cardiotoxicity [59]. Quercetin has a capability to protect the myocardium from cardiac ischemia/reperfusion injury. In this context, it represses the inflammatory cascade and apoptosis, mainly via the PI3K/Akt pathway [60].

Quercetin protected cardiomyocytes from apoptosis, limited myocardial injury from mechanical trauma, and improved cardiac dysfunction via intracellular Ca^2+^ overproduction and the elimination of ROS [61]. However, the accumulated data are scarce and controversial [62]. Quercetin seems to inhibit Ca^2+^ influx via L-type Ca^2+^ channels, leading to myocardium protection against ischemia [63]. Thus, regulation of calcium channels can be a potential cardioprotective mechanism of quercetin which requires further investigation. In parallel, the decrease in ROS production after the treatment with quercetin has also been reported in the model of a 4-hydroxynonenal-induced toxicity H9c2 cardiac tissue-derived cell line. This reduction occurred along with the attenuation of apoptotic stress markers (pMAPKAPK-2, p-SAPK/JNK, p-Hsp27, p-c-JUN, and cl-CASP3), leading to the improvement of cell viability after 24 h quercetin treatment in concentrations ranging from 0.1 to 10 μM [64]. Moreover, H9c2 cells exposed to hypoxia/reoxygenation (H/R) were rescued by quercetin treatment (10–16 μM) [65], while 24 h pretreatment with PLGA-encapsulated quercetin nanoparticles was also preventive [66]. In human cardiomyocytes cultured in H/R medium quercetin can improve the mitophagy mechanism and reduce endoplasmic stress via stimulation of SIRT1/TMBIM6 pathways. Both pathways can increase autophagy and mitophagy events and reverse ROS overproduction induced by hypoxia. Thus, cellular homeostasis can be improved, as well as the vulnerability of cardiomyocytes against H/R events [67]. Similarly, isolated heart models for the ischemia/reperfusion (I/R) injury have shown that heart recovery is improved via the HMGB1 (high mobility group box 1) pathway after 30 min of ischemia followed by 30 min of reperfusion [68]. Moreover, quercetin treatment yielded reduced expression of both TNF-alpha (TNF-α) and interleukin 10 (IL-10) along with their serum levels in rats after acute myocardial ischemia and reperfusion injury [69]. The protective effect of quercetin against adverse remodeling of myocardial infarction has been confirmed in infarction-affected rats treated with 50 mg/kg/orally for 30 days. That effect was attributed to the attenuation of TGF-b1/Smad3 signaling which is associated with cardiac fibrosis, hypertrophy, and cardiac dysfunction [70]. In addition, the treatment of rats with quercetin (10 mg/kg) after a myocardial I/R injury stimulated the phosphoinositide 3-kinases (PI3Ks) pathway, which is responsible for the favorable regulation of cell proliferation, as well as of cellular survival and apoptosis [71].

##### Drug-Induced Cardiotoxicity

Drug-induced cardiotoxicity is one of the most commonly reported adverse effects related with several medicines of different categories. The underlying mechanisms mostly involve drug–protein interactions, which can lead mainly to myocardial infarction or ischemia, heart failure, valvular heart disease, and QT prolongation associated, in several cases, with sudden cardiac death [72]. ROS formation at levels higher than those that can be counterbalanced by cellular detoxification mechanisms causes progressive mitochondrial dysfunction leading to cardiomyocyte apoptosis. Quercetin can scavenge free radicals thanks to the catechol groups in the B ring, the 2,3-double bond in conjugation with the 4-oxo group in the C ring, and the OH groups located at positions 3 and 5 in the heterocyclic ring [73]. ROS deactivation by quercetin takes place in three stages, including formation of a superoxide ion, generation of hydroxyl (or cryptohydroxyl) radicals, and formation of lipid peroxy radicals [74]. Several drugs, such as anthracyclines or protein tyrosine kinase inhibitors, express their cardiotoxicity inducing ROS production and accumulation in cells. Doxorubicin is a chemotherapy drug classified as an anthracycline and employed in different types of cancer [72]. Quercetin given orally to rats before their treatment with daunorubicin showed significant cardiac protection. Moreover, preadministration of the flavonoid combined with angiotensin II receptor blocker losartan can increase the ability of the drug to impede lipid peroxidation and, thus, progression of atherosclerotic plaques. In doxycycline-treated rats, coadministration of these two agents reduced the signs of cardiomyopathy and the induced inflammation [75]. Nazmi et al. (2016) [76] proved the antioxidative and renoprotective activity of quercetin against doxorubicin-induced cardiotoxicity and nephrotoxicity in Wistar albino rats. After the 7-day-long *per os* treatment with 2 mg/kg/day quercetin, the blood glutathione levels restored, while the levels of lipid peroxidation products were reduced. Most importantly, the quercetin-treated rats had no signs of vascular myopathy and featured normal appearance of their myocardium [76]. However, the effect of quercetin on the less cardiotoxic epirubicin was found to be negative for cell viability. In particular, quercetin decreased the elimination of the drug from cardiomyocytes, impeding its transformation to the less toxic secondary alcohol metabolite epirubicinol [77].

##### Metabolic Syndrome (MS)

Metabolic syndrome (MS) is a medical term describing the accumulation of several risk factors increasing the incidence of cardiovascular pathology, such as coronary heart disease, diabetes mellitus, and stroke. The cluster of these risk factors includes hypertension, hyperlipidemia, obesity, and diabetes mellitus [78]. The correlation between the incidence of these factors and quercetin consumption has been extensively investigated in in vitro and preclinical studies performed using models of metabolic syndrome [32,44,79,80] (Table 1). Recently, Hosseini et al. (2021) [5] accumulated the animal studies implicating the beneficial effects of quercetin on metabolic syndrome factors, including T2DM, hypertension, dyslipidemia, and obesity, in their review about quercetin and metabolic syndrome. The given doses in those studies ranged from 5 mg/kg/day to 240 mg/kg/day administered either via intraperitoneal injection or orally (gavage method or via drinking water). The administration period lasted from 10 to 90 days. The studies included either rats or mice, but streptozotocin-induced diabetic rats were the most common animal model used. From the mechanistic perspective, quercetin-induced insulin secretion and insulin sensitization were the principal pathways for the protective effect of quercetin against T2DM [81,82]. Furthermore, quercetin exerted a hepatoprotective action in animal models [83], while the anti-obesity effect of quercetin was associated with its antioxidant and anti-inflammatory activity [84].

The decrease in triglycerides, total cholesterol, and low-density lipoprotein cholesterol has been reported after quercetin administration in several studies [85,86,87]. However, its effect on HDL levels is considered controversial. It has been found substantially increased in apolipoprotein E-deficient mice fed a high-fat diet, whereas a nonsignificant increase was noted in dyslipidemic mice treated with 500 mg/L of quercetin in drinking water [56,87,88]. In the study of Gao et al. (2021) [89], the protective effect of this flavonoid on a nonalcoholic fatty liver disease (NAFLD) rat model was also demonstrated. Taken together, quercetin administration seems to exert a beneficial impact on almost all components of metabolic syndrome simultaneously, presenting a multifactorial cardioprotective agent.

##### Atherosclerosis

Cardiovascular diseases and especially atherosclerosis constitute a complex process influenced by both genetic and environmental factors. There is evidence that quercetin suppresses the progression of atherosclerosis in animal studies, and several anti-atherosclerotic mechanisms have been proposed [53,90]. Quercetin supplementation, alone or combined with exercise, was found to prevent atherosclerotic plaque formation in C57Bl6 LDLr-deficient mice fed an atherogenic diet. This effect is expressed via the stimulation of cholesterol reverse transport genes promoting lipid reduction [91]. A few studies showed atheromatous plaques regression through the induction of autophagy and the delay of senescence, possibly via the activation of the ERK signaling pathway [92,93,94]. A number of in vivo and in vitro studies indicated suppression of the expression of many inflammatory factors, including IL-6, TNF-α, PCSK9, MCP-1, CD36, and others [88,91,95,96,97], as well as upregulation of ABCA1 and LXR-α when quercetin is delivered to mice [88,97]. In the same anti-inflammatory context, quercetin mediates maturation of dendritic cells, inhibition of VCAM-1 and ICAM-1 [98,99,100,101], and inhibition of the galectin–3NLRP3 signaling pathway [102]. Quercetin also seems to confer an atheroprotective effect via the activation of the caspase-3 and NFK-β factors [103], the upregulation of para-oxonase 1 gene expression [104,105], and the suppression of the ER stress-CHOP pathway with a concomitant increase in Bcl-2 gene expression via inhibiting ATF6 and IRE1 activation [106].

Based on mice studies, quercetin has the potential to stabilize atherosclerotic plaques through the inhibition of elastin degradation, macrophage infiltration, MMP-9 and VCfAM-1 expression [106]. Moreover, it can reduce the lipid content, the levels of sICAM-1, IL-6, and VCAM-1 and increase Sirt1 within atheromatous plaques in the aorta of the animal model [107]. Sirt1 is a key molecule for basal-level autophagy [108]. *In vitro*, quercetin blocks the ERK signaling pathway, which in turn inhibits MMP-1 and MMP-9, two important mediators of plaques’ vulnerability [109,110]. From the aforementioned studies, it can be suggested that quercetin has a promising effect on atherosclerotic lesions, but this remains to be confirmed in humans.

Lipid decrease and the suppressive effect of quercetin administration on fat accumulation and the increase in blood lipids has been proposed as an additional antiatherogenic mechanism [111,112,113]. Consequently, lower lipid deposition in macrophages is noted, along with the promotion of cholesterol efflux and blockage of foam cell formation [107]. Furthermore, downregulation of the PCSK9 protein and platelet glycoprotein 4 (CD36) as well as increased expression of the PPARγ and LXRα receptors and the ABCA1 transports in apoE-/- mouse aortas treated with 12.5 mg/kg/day of quercetin were found to be associated with the antiatherogenic effect of the flavonoid. High levels of PCSK9 force the expression of CD36, which is followed by a cascade of events that contribute to the progression of atherosclerotic plaques [114].

##### Obesity

Obesity is associated not only with adipose tissue accumulation, but also with its dysfunction and the occurrence of CVDs [115]. Obesity is an inflammatory process where overexpression of inflammatory cytokines such as TNF-a, IL-6, and MCP-1 is observed. This proinflammatory state leads to endothelial and microvascular dysfunction due to a significant increase in ROS [116]. Quercetin is a CYP2E1 inhibitor capable of neutralizing free radicals and downregulating the inflammatory process, normalizing the main biochemical parameters of blood serum such as the cholesterol, glucose, and creatinine levels [117]. Furthermore, it acts as a blocker of adipogenesis, impeding the mitogen-activated protein kinase (MAPK) signal pathway [118]. Increased levels of the MAPK were found to be related with an increase in body weight and a reduction in energy expenditure [119]. Treatment of 3T3-L1 fibroblast cells with quercetin showed that adipogenesis is downregulated due to the impediment of extracellular signal-regulated kinases’ (ERK) and c-Jun N-terminal kinases’ (JNK) phosphorylation and the activation of adipocyte apoptosis [118]. In their work on C57B1/6 mice, Jung et al. (2012) [111] proved the anti-obesity effect of quercetin on high-fat diet-induced obesity, and this effect was attributed to its involvement in lipogenesis and lipid metabolism.

### 3.4. Quercetin and Cardiovascular Prevention Based on Clinical Studies

Cardiovascular diseases (CVD) are the main cause of morbidity and mortality worldwide, with atherosclerosis being considered the underlying mechanism [122]. Traditional cardiovascular risk factors including hypertension, dyslipidemia, diabetes, and obesity often coexist and share common pathophysiologic mechanisms [123]. Certain antihypertensive, antithrombotic, anti-dyslipidemic, and anti-atherosclerotic properties of quercetin have been proposed for CVD protection [124]. Clinical studies and meta-analyses summarized in Table 2 and Table 3 evaluated the impact of quercetin on the cardiovascular risk factors described below.

#### 3.4.1. Metabolic Syndrome (MS)

A recent systematic review [125] of seventeen RCTs evaluated the effect of daily supplementation with quercetin in patients with MS, including hypertension, T2DM, PCOS, and obesity, as well as in healthy individuals. The dosage of quercetin ranged from 30 to 1000 mg/day and the duration of intervention varied between 2 and 12 weeks along the studies. The cumulative data demonstrated significantly reduced both systolic and diastolic blood pressure. Although the previous systematic review failed to report any significant change in lipid or glucose profiles after the administration of quercetin, subgroup analysis showed significant amelioration of HDL and triglycerides concentrations in trials where the duration of quercetin administration exceeded 8 weeks. Another meta-analysis [126] of eighteen RCTs assessed the effect of flavonol supplementation for 2 to 12 weeks on MS parameters, including plasma lipids, glucose, and blood pressure. Significant decrease was observed in blood pressure, fasting glucose, LDL, and total cholesterol, as well as a significant increase in HDL cholesterol levels. However, those results should be interpreted with caution because of the studied population. Most of the selected studies (*n* = 13 studies) enrolled healthy subjects, while the rest recruited patients with MS (*n* = 4 studies). Notably, only one study included patients with hypertension. Among the selected RCTs, nine used pure quercetin supplementation, while the rest (*n* = 9 studies) investigated interventions of either pure flavonol supplements or enriched mixtures of flavonols. Most recently, an observational prospective study of 6417 participants [127] showed that a higher dietary intake of flavonoids (combination of quercetin with magnesium) was associated with a lower incidence of MS. Similarly, a recent RCT [128] confirmed the favorable effects of quercetin administration on such MS parameters as body weight, cholesterol metabolism, fasting plasma insulin, and blood pressure. In the context of MS, quercetin may additionally protect against the associated liver disease [129]. After all, the up-to-date data are conflicting, and there is no robust evidence that supplementary quercetin treatment effectively modifies MS parameters.

#### 3.4.2. Obesity

A recent meta-analysis [125] of nine RCTs evaluated the effects of quercetin administration on body weight. Overall, supplementation with quercetin had no significant impact on body weight, BMI, waist circumference, or waist-to-hip ratio among the participants with obesity, hypertension, and PCOS. Pfeuffer et al. (2011) [130] introduced the influence of genetic polymorphism on quercetin’s efficacy since they showed significantly decreased BMI, body weight, and waist circumference in APOE3/3 but not in APOE4 patients. The association between quercetin and APOE isoforms was observed in another study [131] of overweight/obese subjects with MS. Moreover, the APOE3 group, but not the APOE4 group, responded to quercetin therapy with a decrease in systolic blood pressure. On the other hand, significant reductions in plasma-oxidized LDL and TNF-a were observed in both groups.

#### 3.4.3. Hypertension

There are controversial results about the impact of quercetin supplementation on blood pressure. A systematic review [132] of seven RCTs demonstrated significant reductions in both systolic and diastolic blood pressure after quercetin supplementation in patients with hypertension [133]. It seems like the hypotensive effects of quercetin are more evident with a higher dosage (>500 mg/day) and in subjects with certain characteristics, including MS and smoking [125,132,133]. Another recent meta-analysis [134] of eight RCTs showed a significant decrease in systolic blood pressure only and not in diastolic blood pressure following supplementation with quercetin in patients with MS. In contrast, a recent large (*n* = 15,662 participants) prospective cohort study [135] failed to show any significant association between quercetin dietary intake as a measure of primary prevention and the incidence of hypertension [135]. In line, other eight clinical studies [9,131,136,137,138,139,140,141] conducted between 1998 and 2019 failed to find any significant effect of quercetin supplementation on blood pressure in healthy and hypertensive subjects, while other cardiovascular risk factors (lipid levels, inflammatory mediators, or thrombogenic risk factors) remained unaffected as well. Presumably, the validity of the latter studies is limited since they enrolled a small number of participants, had a short duration of intervention, and the majority of the participants were healthy individuals or patients with prehypertension or, at maximum, stage I hypertension.

#### 3.4.4. Dyslipidemia

In a recent meta-analysis by Sahebkar et al. (2017) [142], quercetin supplementation significantly reduced plasma TG at doses above 50 mg/day. However, available evidence from the five RCTs included in that meta-analysis showed a nonsignificant relationship between quercetin supplementation and the rest of the lipid parameters (total cholesterol, LDL, HDL). Another systematic review of nine RCTs, by Guo et al. (2019) [143], failed to find any significant change in plasma lipids after quercetin treatment unless higher doses of quercetin (≥250 mg/d) were administered to overweight and obese individuals which could reduce LDL cholesterol. Hence, the pure effect of quercetin on lipids remains controversial.

Other investigators have supported the favorable effects of quercetin in dyslipidemic patients when used as an adjunctive therapy or in patients with MS. In particular, Mazza et al. (2021) [144] showed improved TG and LDL plasma levels in dyslipidemic and hypertensive patients with statin intolerance when receiving quercetin in combination with ezetimibe. A recent meta-analysis of sixteen RCTs (Tabrizi et al., 2020) [145] indicated that quercetin supplementation significantly decreased plasma levels of total cholesterol, LDL, and CRP in patients with MS. However, it did not affect the levels of TG, HDL, IL-6, and TNF-α. Furthermore, two more studies [146,147] reported that consumption of quercetin-rich onion juice or powder significantly reduced plasma lipids and prevented obesity. It is undoubtable that these beneficial effects differ significantly depending on the formulation and dosage of quercetin, as well as the duration of administration.

#### 3.4.5. Hyperglycemia

In a recent systematic review (Ostadmohammadi et al., 2019) [148] of nine RCTs, quercetin supplementation did not affect fasting glucose or hemoglobin A1 levels or insulin resistance in patients with MS including hypertension, T2DM, PCOS, or obesity. The duration of intervention varied between 4 and 12 weeks and the dosage of quercetin ranged from 100 to 1000 mg/day. However, subgroup analysis revealed that longer duration (≥8 weeks) and higher dosages of quercetin (≥500 mg/day) were significantly correlated with reduced fasting plasma glucose. Similarly, higher doses of quercetin (≥500 mg/day) effectively decreased insulin levels only in studies recruiting younger subjects (aged <45 years old). A large prospective study by Song et al. (2005) of 38,018 healthy women failed to demonstrate a preventive effect of dietary flavonoids, including quercetin, on T2DM [149]. In contrast, a very small noncontrolled pilot study of 15 T2DM patients who for 3 months received *Eugenia punicifolia*, mainly containing quercetin, showed a significant decrease in hemoglobin A1 and basal insulin [150]. Further large-scale trials are needed to investigate the impact of quercetin on T2DM management and prevention.

The inconsistent data about the impact of quercetin on cardiometabolic risk factors highlights the need for further human studies.

#### 3.4.6. Quercetin and CVDs

Recent evidence has emerged that changes in gut microbiota are related to the occurrence of chronic low-grade inflammation and, thus, to the development of CVDs, including coronary artery disease, heart failure, and hypertension. Quercetin’s dose-dependent effects on the gut microbiota have an impact on CVD progression [151,152]. Quercetin-like natural plant compounds usually affect multiple targets to prevent cardiovascular events. Thus, the activity of quercetin-like plant compounds as cofactors of COX-2 is just one mechanism by which they decrease the risk of cardiovascular diseases, and more research is needed to confirm this hypothesis [153]. Endothelial dysfunction is an important step seen early in the progression of atherosclerosis and hypertension [154].

A randomized controlled trial investigated the effect of quercetin (120 mg per day for 2 months) on 30 patients with coronary artery disease and showed a positive effect on chronic systemic inflammation indicated by reduction in IL-1β and the IkBa gene in blood mononuclear cells [155]. In a cohort of 5133 healthy adults, lower dietary intake of flavonoids, including quercetin, was correlated with higher risks of coronary artery disease [156]. Quercetin administration is generally well-tolerated, with some minor side effects such as mild headache, nausea, and tingling of the extremities observed in long-term supplementation at 1000 mg/day. Nephrotoxicity has been reported with high IV doses in cancer patients [154].

**Table 2:** Published meta-analyses of randomized clinical trials investigating the impact of quercetin on classical cardiovascular risk factors.

Reference	Studies and Cohorts	Study Design	Outcomes
Huang et al., 2019 [130]	Nine RCTs, 525 pts; obese, HTN, PCOS, healthy individuals	Quercetin daily; dose: 100–1000 mg; duration: 2–12 weeks	↔ body weight, BMI, waist circumference, waist-to-hip ratio
Huang et al., 2020 [125]	17 RCTs, 896 pts; MS, T2DM, PCOS, obesity	Quercetin daily; dose: 30–1000 mg; duration: 2–12 weeks	↓ SBP and DBP ↔ lipid and glucose profile Subgroup analysis (> 8 weeks after intervention): ↑ HDL, ↓ TG
Menezes et al., 2017 [126]	18 RCTs, 530 pts; healthy individuals, MS, HTN	Flavonol daily; dose: 16–1200 mg; duration: 2–12 weeks	↓ BP, FPG, LDL, Tchol, triacylglycerol ↑ HDL Subgroup analysis: favorable in dyslipidemic pts and pts of Asian origin
Ostadmohammadi et al., 2019 [150]	Nine RCTs, 781 pts; MS	Quercetin daily; dose: 150–1000 mg; duration 4–12 weeks	↔ FPG, HbA1c, insulin resistance
Sahebkar et al., 2017 [144]	Five RCTs, 442 pts; central obesity, hypertriglyceridemia, T2DM, HTN	Quercetin daily; dose: 30–730 mg; duration: 2–10 weeks	↓ TG (at doses > 400 mg/day) ↔ Tchol, LDL, HDL
Serban et al., 2016 [133]	Seven RCTs, 587 pts; HTN	Quercetin daily; dose: 100–1000 mg; duration: 4–10 weeks	↓ SBP and DBP
Tabrizi et al., 2020 [147]	16 RCTs, 1575 pts; MS	Quercetin daily; dose: 3.12–3000 mg; duration: 2 h postprandially for 12 weeks	↓ Tchol, LDL, CRP ↔ TG, HDL, IL-6, TNF-α

BMI, body mass index; CRP, C-reactive protein; DBP, diastolic blood pressure; FPG, fasting plasma glucose; HDL, high-density lipoprotein; HTN, hypertension; IL-6, interleukin 6; LDL, low-density lipoprotein; MS, metabolic syndrome; PCOS, polycystic ovarian syndrome; pts, participants; RCTs, randomized controlled trials; SBP, systolic blood pressure; T2DM, type 2 diabetes mellitus; Tchol, total cholesterol; TG, triglycerides; TNF-α, tumor necrosis factor-α; wks, weeks; ↓, decrease; ↑, increase; ↔, non-significant change.

**Table 3:** Published clinical studies investigating the impact of quercetin on the classical cardiovascular risk factors not included in meta-analyses.

Reference	Study Cohort/and Condition	Study Design	Outcomes
Biesinger et al., 2016 [138]	18 pts; MS	Crossover RCT; quercetin dehydrate daily vs. placebo; dose: 25 mg; duration: 28 days; washout period: 2 weeks	↔ HTN incidence
Brull et al., 2015 [141]	68 pts; MS, overweight or obese, prehypertension and stage I HTN	RCT, crossover study; quercetin from onion skin extract daily vs. placebo; dose: 162 mg; duration: 6 wks; washout period: 6 weeks	↔ HTN incidence, endothelin-1, ADMA, ACE activity, CRP, endothelin-1, sE-selectin, sVCAM-1, sICAM-1, RHI, AI
Burak et al., 2019 [143]	67 healthy nonobese volunteers aged 19–35 years	Randomized double-blind placebo-controlled crossover study; 3.6 g/day ALA plus 190 mg/day quercetin vs. placebo; duration: 8 wks; washout period: 8 weeks	↔ office systolic BP, mean 24-hour ambulatory BP, mean ambulatory BP, HDL, apolipoprotein A1, glucose, uric acid, oxidized LDL, CRP
Chekalina et al., 2018 [155]	85 pts; CAD	RCT; intervention group (30 pts): quercetin, 120 mg daily; control group (55 pts): placebo; duration: 2 mo	↓ IL-1b, TNF-α, expression of the IkBa gene in blood mononuclear cells
Conquer et al., 1998 [9]	27 pts; healthy	RCT; intervention group (13 pts): quercetin, 1000 mg daily	↔ HTN incidence, Tchol, LDL, HDL, TG, platelet aggregation, platelet thromboxane B2 production, resting heart rate
Edwards et al., 2007 [139]	41 pts; prehypertension and stage I HTN	Crossover RCT; quercetin aglycone twice/day vs. placebo; dose: 365 mg; duration: 4 weeks; washout period: 2 weeks	↔ HTN incidence, weight, BMI, indices of oxidative stress, TG, LDL, VLDL, HDL, Tchol, FPG Prehypertensive subjects: ↓ SBP, DBP, MAP
Egert et al., 2009 [137]	93 pts; overweight or obese pts, MS	Double-blind placebo-controlled crossover study; quercetin daily vs. placebo; dose: 150 mg; duration: 6 weeks; washout period: 5 weeks	↓ HDL, oxidized LDL ↔ incidence of HTN, Tchol, TG, LDL:HDL cholesterol, TG:HDL cholesterol ratios, TNF-α, CRP
Egert et al., 2010 [132]	93 overweight/obese pts; MS, APOE3/3, 3/4, 4/4, 2/3, 2/4	Double-blind placebo-controlled crossover study; quercetin daily vs. placebo; dose: 150 mg; duration: 6 weeks; washout period: 5 weeks	↓ oxidized LDL, TNF-α ↔ CRP, body weight, waist circumference, fat mass, fat-free mass APOE3/3 group: ↓ SBP APOE4 group: ↓ HDL
Jin et al., 2021 [127]	6417 subjects	Observational prospective cohort study; dietary intake flavonoids (quercetin + magnesium)	↓ incidence of MS
Knekt et al., 1996 [156]	5133 healthy adults aged 30–69 years	Cohort study; dietary intake of flavonoids	↓ incidence of CAD
Larson et al., 2012 [142]	17 men; normotensive and stage I HTN	Double-blind placebo-controlled crossover study; quercetin aglycone (dose: 1095 mg) vs. placebo; duration: acute single dose	Normotensive: ↔ BP, ACE activity Stage I HTN: ↓ SBP, DBP, mean BP ↔ ACE activity, NO metabolites, ET-1, ET-1:NO ratio metabolites
Lu et al., 2015 [148]	24 healthy subjects; mild hypercholesterolemia	Pilot RCT; intervention group (12 pts): 100 ml quercetin-rich onion juice daily; control group (12 pts): placebo; duration: 8 weeks	↓ waist circumference, Tchol, LDL
Mazza et al., 2021 [146]	96 pts; dyslipidemia, HTN, statin intolerance	RCT; intervention group (48 pts): ezetimibe/quercetin, 10/100 mg daily; control group (48 pts): ezetimibe monotherapy; duration: 3 mo	↓ TG, LDL
Nishimura et al., 2020 [149]	70 healthy subjects	RCT; intervention group (35 pts): 9 g quercetin-rich onion powder daily; control group (35 pts): placebo; duration: 12 weeks	↓ ALT ↔ visceral fat area, BP
Pfeuffer et al., 2013 [131]	49 healthy men; APOE3/3, 3/4, 4/4	Double-blind crossover study; quercetin daily vs. placebo; dose: 150 mg; washout period: 3 weeks	↔ endothelial function APOE3/3 group: ↓ BMI, body weight, waist circumference
Sales et al., 2014 [152]	15 pts; T2DM	Pilot study; intervention: capsules containing 200 mg dried leaves of E. punicifolia; duration: 3 mo	↓ HbA1c, basal insulin, thyroid-stimulating hormone, CRP, SBP, DBP
Shatylo et al., 2021 [128]	110 pts aged >60 years; MS	RCT; intervention group (55 pts): 240 mg quercetin daily; control group (55 pts): placebo; duration: 3 mo	↓ SBP and DBP, body weight, BMI, Tchol, LDL, insulin, 2-hour glucose level ↔ HDL, TG, oxidative stress (CAT, SOD, AGEs)
Song et al., 2005 [151]	38,018 healthy women aged >45 years	Cross-sectional study; dietary intake of flavonoids	↔ incidence of T2DM
Yao et al., 2021 [136]	15,662 subjects	Prospective cohort study; dietary intake of quercetin daily	↔ HTN incidence
Zahedi et al., 2013 [140]	62 women aged 35–55 years; T2DM	RCT; intervention group (34 pts): quercetin, 500 mg daily; control group (28 pts): placebo; duration: 10 wks	↓ SBP, HDL ↔ HTN incidence, DBP, Tchol, LDL, TG, ratio of TG/HDL and LDL/HDL, TNF-α, IL-6, hs-CRP

ACE, angiotensin-converting enzyme; ADMA, asymmetric dimethylarginine; AGEs, advanced glycoxidation end products; AI, augmentation index; ALT, alanine transaminase; APOE, apolipoprotein E; BMI, body mass index; CAD, coronary artery disease; CRP, C-reactive protein; DBP, diastolic blood pressure; FPG, fasting plasma glucose; HDL, high-density lipoprotein; HTN, hypertension; IL, interleukin; LDL, low-density lipoprotein; mo, months; MS, metabolic syndrome; PCOS, polycystic ovarian syndrome; RCTs, randomized controlled trials; RHI, reactive hyperemia index; SBP, systolic blood pressure; sICAM-1, soluble intercellular adhesion molecule 1; sVCAM-1, soluble vascular cell adhesion molecule 1; T2DM, type 2 diabetes mellitus; Tchol, total cholesterol; TNF-α, tumor necrosis factor-α; TG, triglycerides; wks, weeks; ↓, decrease; ↑, increase; ↔, non-significant change.

## 4. Study Limitations

The published data are quite promising; however, there are several drawbacks before considering the net results of the effects of quercetin on cardiovascular risk factors and diseases. The mechanistic explanations are derived from mostly in vitro rather than in vivo studies. Despite the growing number of studies, the underlying mechanisms of quercetin action remain unclear or somehow speculative and require further investigation using appropriate animal models. Most of the clinical studies summarized in Table 3 are of small sample size, while quercetin was administered as a plant extract or within the dietary intake of flavonoids as a food supplement. Furthermore, the dose administered varied from 3.12 mg to 3000 mg daily, while the clinical studies also differed with respect to study duration and patients’ characteristics. It should also be mentioned that literature lacks pharmacokinetic/pharmacodynamic and dose linearity studies of quercetin that are essential in the optimum design of clinical trials before any clinical application of quercetin and significantly compromises the validity and reproducibility of the clinical studies.

## 5. Conclusions

Flavonol quercetin is the main pharmacologically active ingredient of many fruits, vegetables, tea, and red wine with antioxidant, anti-inflammatory, hypotensive, glucose- and lipid-lowering activity. A part of those observations is also supported by results of the existing clinical studies that have shown an improvement in glycemic and lipid profile. However, the inconsistent data about the impact of quercetin on cardiometabolic risk factors such as MS, obesity, and hypertension point out the need for further human studies. Accordingly, additional properly designed clinical trials for specific cardiac conditions using standard dosages based on the previously performed pharmacokinetic/pharmacodynamic studies are required to test its value as a food supplement to conventional drug therapies.

## Figures and Tables

**Table 1:** Published preclinical studies investigating the impact of quercetin on classical cardiovascular risk factors.

Reference	Animal Model	Study Design	Outcomes
Alam et al., 2014 [45]	Swiss albino mice	20 mg/kg/day quercetin orally for 3 weeks	↓ FBG ↑ GLUT-4
Albadrani et al., 2020 [70]	Wistar albino rats	50 mg/kg/day quercetin orally for 4 weeks	↓ TGF-b1/Smad3 signaling
Abdelkarem and Fadda, 2017 [44]	Sprague–Dawley rats	50 mg/kg/day quercetin orally for 4 weeks	↓ serum glucose, TC, LDL-C, TG, leptin, adiponectin
Carlstrom et al., 2007 [40]	Spontaneously hypertensive rats (SHR) and Wistar Kyoto rats (WKY)	1.5 g quercetin/kg/day quercetin orally (gavage) for 5 or 11 weeks (SHR)	↔ BP, cardiac hypertrophy, vascular dysfunction, vascular remodeling, and indices of oxidative stress in SHR
Castillo et al., 2018 [54]	Wistar albino rats	HC diet supplemented with 0.5% *w/w* quercetin for 4 weeks	↓ TG, TC, glucose, oxidative stress suppression, LDL-C and VLDL-C increase, HDL-C decrease
Cui et al., 2017 [56]	apoE−/− mice	12.5 mg/kg/day quercetin via gavage	↑ RCT
Duarte et al., 2009 [39]	Spontaneously hypertensive rats (SHR) and normotensive Wistar Kyoto rats (WKY)	10 mg/kg/day quercetin orally (gavage) for 5 weeks	↓ SBP, DBP, and MAP in SHR
Elbarbry et al., 2020 [38]	Spontaneously hypertensive rats (SHR)	10, 30, and 60 mg/L quercetin in drinking water for 7 weeks	↓ MAP at a high dose of quercetin
Gao et al., 2021 [89]	Nonalcoholic fatty liver disease (NAFLD) rat model	80, 40, or 20 mg/kg/day quercetin via gavage for 4 weeks	↓ TC, TG, blood glucose levels
Garelnabi et al., 2022 [91]	C57BL6 LDLr−/− mice	100 μg/day quercetin orally for 4 weeks	↓ AP, MCP-1
Gomes et al., 2015 [46]	apoE−/− mice	10 mg/kg/day quercetin orally for 4 weeks	↓ plasma glucose, TG, TC, tendency to reduce proteinuria and glomerular injury
Häckl et al., 2002 [41]	Wistar albino rats	Oral or IV preadministration of 88.7 μmol/kg and 14.7 μmol/kg quercetin, respectively, for 45 and 5 min before a bradykinin IV injection	↑ hypotensive effect of bradykinin ↓ MAP
Hemmati et al., 2018 [120]	Wistar albino rats	15 mg/kg/day quercetin (i.p. injection) for 3 weeks	↓ MDA, mRNA levels of HSP27, HSP70, HSF-1, and glucose-6-phosphatase ↑ glucokinase expression
Iwara et al., 2022 [79]	Albino rats	10 mg/kg/day quercetin orally for 3 weeks	↓ FBG ↑ BW, AST, ALP, ALT, albumin
Jia et al., 2019 [88]	apoE−/− mice	12.5 mg/kg/day quercetin orally for 12 weeks	↓ TC, LDL-C, oxLDL, TNF-α, IL-6, plaques ↑ IL-10, PPAR-γ, LXRα ABCA1
Jin et al., 2012 [96]	Sprague–Dawley rats	1 mg/kg quercetin IV	↓ TNF-α, IL-10
Jung et al., 2012 [111]	C57BL/6J mice	HC diet supplemented with 0.025% *w/w* quercetin for 9 weeks	↓ BW, size of the epididymal adipose tissue and liver tissue, TBARS, fat, altered expression of the lipid metabolism-related genes
Juźwiak et al., 2005 [53]	Mongrel rabbits	0.05 mg/kg/day quercetin orally for 4 and 12 weeks	↓ TG, TC, plaque formation, thickening of the tunica intima of the aorta
Kuipers et al., 2018 [85]	C57Bl/6J mice	HC diet supplemented with 0.1% *w/w* quercetin for 12 weeks	↓ TG, white adipose tissue browning
Le et al., 2014 [121]	C57BL/6J mice	HC diet supplemented with 0.05% and 0.1% *w/w* quercetin for 9 weeks	↓ TNF-α, MCP-1 skeletal muscle atrophy
Liang et al., 2021 [58]	Hypercholesterolemia hamsters	2.5 g/kg/day quercetin orally for 8 weeks	↔ TC
Lin et al., 2020 [30]	Spontaneously hypertensive rats (SHR)	10 mg/kg/day quercetin orally (gavage) for 6 weeks	↓ SBP, DBP ↑ autophagy
Mariee et al., 2012 [83]	Sprague–Dawley rats	15 mg/kg/day quercetin orally for 2 weeks	↓ TG, TC, LDL-C, ALT, AST, γ-GT, liver TBARS ↑ HDL-C, GSH
Matouk et al., 2013 [75]	Wistar albino rats	10 mg/kg/day quercetin orally for 4 weeks	↓ TNF-α, LDH, CK-MB, MDA, NO ↓ CAT, ↓SOD
Muselin et al., 2022 [87]	BALB/c mice	500 mg/L quercetin in drinking water (duration of the study not reported)	↓ TC, LDL-C, TG
Nazmi et al., 2016 [76]	Wistar albino rats	2 mg/kg/day quercetin orally for 1 week	↑ AST, LDH, BUN, creatinine, GSH
Pereira et al., 2018 [32]	2K1C hypertensive Wistar albino rats	10 mg/kg/day quercetin via gavage for 3 weeks	↓ SBP, BW, ROS, MLP
Rasheed et al., 2022 [113]	Albino rats	50 mg/kg/day of quercetin orally for 12 weeks	Improvement of the histopathological degenerative and inflammatory changes ↓ mean area % of collagen fibers
Ting et al., 2018 [84]	Wistar albino rats	13 mg/kg/day quercetin orally for 8 weeks	↓ BW, ALT, TG, TC, size of perirenal adipocytes ↑ adiponectin expression, AST
Wang et al., 2013 [71]	Sprague–Dawley rats	10 mg/kg quercetin (i.p. injection) 5 min before reperfusion	↓ infarct size, serum levels of creatine kinase and lactate dehydrogenase, caspase-3 immunoreactivity, and Bax expression ↑ Akt phosphorylation and Bcl-2 expression
Zhou et al., 2021 [80]	Sprague–Dawley rats	Oral preadministration of 5 and 10 mg/kg quercetin	↓ TG, fat absorption ↑ fat excretion

ALP, alkaline phosphatase; ALT, alanine aminotransferase; AP, atherosclerotic plaques; AST, aspartate aminotransferase; BP, blood pressure; BUN, blood urea nitrogen; BW, body weight; CAT: myocardial catalase; CK-MB, creatine kinase; DBP, diastolic blood pressure; FBG, fasting blood glucose; GLUT-4, glucose transporter type 4; GSH, glutathione; HC, high cholesterol; HDL, high-density lipoprotein; HR, heart rate; HSF1, heat shock factor; 1HSP27, heat shock protein 27; HSP70, heat shock protein 70; IL-10, interleukin 10; i.p., intraperitoneal; IV, intravenously; LDH, lactate dehydrogenase; LDL-C, low-density lipoprotein cholesterol; LXRα, liver X receptor alpha; MAP, mean arterial pressure; MCP-1, monocyte chemoattractant protein-1; MDA, malondialdehyde; MLP: myocardial lipid peroxidation; oxLDL, oxidative modification of a low-density lipoprotein; PPAR-γ, peroxisome proliferator-activated receptor-γ; RCT, reverse cholesterol transport; ROS, reactive oxygen species; SBP, systolic blood pressure; SOD, superoxide dismutase; TBARS, thiobarbituric acid reactive substances; TC, total cholesterol; TG, triglycerides; TGF-b1, transforming growth factor beta 1; TNF-α, tumor necrosis factor; VLDL-C, very low-density lipoprotein cholesterol; ↓, decrease; ↑, increase; ↔, non-significant change.

## Data Availability

Data sharing not applicable.
